# At times, myomectomy is mandatory to effect delivery

**DOI:** 10.1186/1750-1164-5-9

**Published:** 2011-10-28

**Authors:** Rajiv Mahendru, Parneet Kaur Sekhon, Geetinder Gaba, Shweta Yadav

**Affiliations:** 1 Dept. of Obs & Gyn, M.M.I.M.S.R.,Mullana, Ambala, Haryana, India

**Keywords:** pregnancy, leiomyoma, myomectomy

## Abstract

**Background:**

Excision of a leiomyoma has never been a choice during caesarean section.

**Method:**

Myomectomy of a massive fibroid was necessitated prior to delivering the baby.

**Results:**

Delivery of a healthy was effected by Classical caesarean section.

**Conclusion:**

Leiomyoma in pregnancy is not an unknown entity and is a cause of concern for being a source of excruciating pain, at times, during the ongoing gestation.

Although performed rarely, it is sometimes necessary to remove a large myoma to effect delivery of the baby during Cesarean section as is depicted in the case being presented hereunder.

## Introduction

According to the various studies, incidence of myomas in pregnancy is estimated to be 2-4%[1-3]. Albeit myomectomy performed during pregnancy remains a rarity, increasing rate of myomectomies during cesarean section has been reported in the past decade[4-6] and, above all, certain studies have regarded this as an effective and safe procedure that is not associated with much bleeding or other complications[7].

## Case report

Written informed consent was taken and the department Ethical Committee approved the case report.

A thirty year aged primigravida reported in the out-patient department of our rural based institute at 14 weeks of pregnancy for routine ante-natal check-up. Her per-abdomen findings revealed uterine size to be inconsistently increased to 22 weeks with the patient being sure of her date of last menstrual period and no history of 'quickening' as yet. Her routine investigations were normal but sonography revealed 13.4 weeks intrauterine live fetus along with a large 11.5 cm intramural leiomyoma on the anterior wall of the maternal uterus encroaching upon the cervix.

The patient was regularly followed up and twice she had had to be admitted, for a couple of days each, in the second trimester with pain abdomen and was managed conservatively. A detailed sonographic image of the fetus at 20 weeks of gestational age showed no evidence of any major structural abnormalities. During the course of pregnancy, this myoma showed progressive increase in size and the last ultrasonography at term depicted it to be 31.3 cm in its longitudinal dimension and involved not only the most of the anterior uterine portion but was also abutting on the lower uterine segment. The presenting part (cephalic) was visualized high above the internal os with this leiomyoma intervening in between. She also had pregnancy induced hypertension of and was treated with oral labetalol from 31.5 weeks onwards and the fetus showed signs of intra-uterine growth restriction.

Pelvic examination, at term gestational age, confirmed the virtual improbability of vaginal delivery. After discussion with the experienced senior sonologist, the patient and her attendants, decision for performing caesarean section was forthcoming. Also the consent for cesarean hysterectomy was sought in case of any eventuality, whatsoever.

At the time of surgery, patient's hemoglobin reading was 10.4 g/dl, nevertheless, four units of cross-matched blood were arranged.

Right sided paramedian longitudinal incision was employed which was extended supra-umbilically. The upper uterine segment was incised longitudinally followed by enucleation of a mammoth sized myoma en masse, that is, classical cesarean section with a myomectomy had to be resorted to. A live female baby weighing 2,280 g was successfully delivered with Apgar scores of 8 and 9 at 1 min and 5 min, respectively.

**Figure 1:**
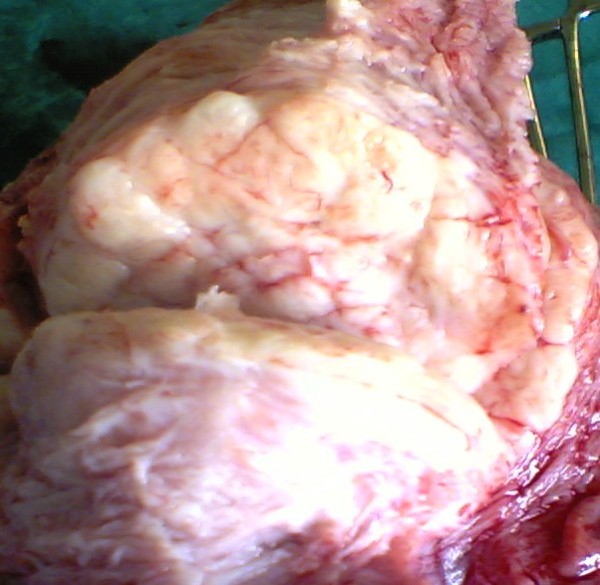
**A large leiomyoma during cesarean section**.

Incised uterine wall was sutured in three layers to obliterate any physiological dead space. Neither uterine vessels were clamped nor vasopressors were used during the intra-operative period. The myoma removed, (Figure 1), was of the dimensions 33.3 × 28.8 × 15.6 cm and the intra-operative blood loss was estimated to be 1860 ml. Two units of cross-matched blood were transfused - one intra-operatively and the other one in the post-operative ward and hemoglobin reading was 9.6 g/dl on the second post-operative day.

Her post-operative recovery was uneventful and she was discharged, along with her baby, on the seventh day after surgery in a satisfactory condition.

## Discussion

The uterine leiomyomas during pregnancy with sufficient tumor size, most often, are clearly detected by the ultrasound examination[2]. Although the reported incidence varies widely, prevalence of fibroids, during pregnancy, larger than 1 cm is mentioned to be 1.6%[8], while in a study conducted by Exacoustos and Rosati[9], 4% patients were diagnosed of having leiomyomas > 3 cm. Vergani et al[2] followed 25,154 pregnant women, where 608 cases (2.4%) had a myoma in the uterus with the size being < 5 cm in 342 cases and ≥ 5 cm in the remaining pregnant women.

Almost 22-32%^3 ^of uterine leiomyomas may progressively increase in diameter owing to persistent stimulation by estrogen[4] and the profuse blood supply during pregnancy. After reviewing previous studies, the antepartum complication rate varied between 10 and 40%[3] and one complication of pain attributable to red degeneration was noted in our patient for which she was admitted twice.

Difficulties encountered at the event of delivery could be due to dysfunctional labor, type of delivery, and morbidity and mortality rates of the fetus and motherwith the possibility of an excessively large tumor inducing uterine atony during labor[2,3]. If the location of the leiomyoma is in the lower segment of the uterus, it may block the passage required for a vaginal delivery as was the case in our patient. A cesarean section may be suggested by obstetricians before labor onset after a prenatal evaluation and the same was followed in the case under study. In a retrospective cohort study[2] consisting 251 pregnant women with a large leiomyoma (≥ 5 cm), it was found that the risk of a cesarean delivery before labor might be enhanced by 26% for each 1-cm diameter increase in the myoma.

After reviewing the literature, suggestion was against performing an intrapartum myomectomy because of increased hemorrhage unless the fibroid was pedunculated or subserosal[3]. Hassiakos et al[5] reported on 47 pregnant women who had a myomectomy during cesarean section, and another 94 women who had leiomyomas but only underwent a cesarean delivery and found that the myomectomy group showed only a mild increase in the operative time (mean, 15 minutes) with no other intra-operative or puerperal complications and the latter was also evident in our case. A study by Kaymak et al[4] showed no differences except for a small increase in the duration of the operation (9 minutes) and the length of hospital stay (0.6 days) in the cesarean myomectomy. Yuddandi[6] reported a successful cesarean section with removal of a massive fibroid of 17 cm in diameter and inspite of a excessive total discharge during the surgery, the patient returned home 1 week later without any complications. Similar success was achieved in the current case being discussed and this is consistent with the case-control study conducted by Li et al[7].

## Conclusion

At times myomectomy becomes inevitable during cesarean delivery but, nonetheless, the emphasis should be that it is to be performed by an obstetrician experienced enough in handling the amount of intraoperative hemorrhage with a very quick technique.

## Conflicts of interests

The authors declare that they have no competing interests.

## Authors' contributions

RM conducted the surgery, conceived, drafted, scripted and edited the manuscript; PKS drafted the manuscript, GG drafted the manuscript; SY assisted in editing the manuscript. All authors have read and approved the final manuscript.
